# Ciguatoxin-Producing Dinoflagellate *Gambierdiscus* in the Beibu Gulf: First Report of Toxic *Gambierdiscus* in Chinese Waters

**DOI:** 10.3390/toxins13090643

**Published:** 2021-09-10

**Authors:** Yixiao Xu, Xilin He, Wai Hin Lee, Leo Lai Chan, Douding Lu, Pengbin Wang, Xiaoping Tao, Huiling Li, Kefu Yu

**Affiliations:** 1 Key Laboratory of Environment Change and Resources Use in Beibu Gulf, Ministry of Education, Nanning Normal University, Nanning 530001, China; xuyixiao_77@163.com (Y.X.); hexilin18177140136@163.com (X.H.); taoxiaoping0613@163.com (X.T.); huiling18307713906@163.com (H.L.); 2 Guangxi Key Laboratory of Earth Surface Processes and Intelligent Simulation, Nanning Normal University, Nanning 530001, China; 3 Guangxi Laboratory on the Study of Coral Reefs in the South China Sea, Guangxi University, Nanning 530004, China; 4 The State Key Laboratory of Marine Pollution, Department of Biomedical Sciences, City University of Hong Kong, Hong Kong, China; waihlee-c@my.cityu.edu.hk (W.H.L.); leochan@cityu.edu.hk (L.L.C.); 5 Shenzhen Key Laboratory for the Sustainable Use of Marine Biodiversity, Research Centre for the Oceans and Human Health, City University of Hong Kong Shenzhen Research Institute, Shenzhen 518057, China; 6 Key Laboratory of Marine Ecosystem Dynamics, Second Institute of Oceanography, Ministry of Natural Resources, Hangzhou 310012, China; doudinglu@sio.org.cn (D.L.); algae@sio.org.cn (P.W.); 7 The Fourth Institute of Oceanography, Ministry of Natural Resources, Beihai 536000, China

**Keywords:** *Gambierdiscus*, morphology, phylogeny, ciguatoxin, benthic dinoflagellate, Beibu Gulf, Chinese waters

## Abstract

Ciguatera poisoning is mainly caused by the consumption of reef fish that have accumulated ciguatoxins (CTXs) produced by the benthic dinoflagellates *Gambierdiscus* and *Fukuyoa*. China has a long history of problems with ciguatera, but research on ciguatera causative organisms is very limited, especially in the Beibu Gulf, where coral reefs have been degraded significantly and CTXs in reef fish have exceeded food safety guidelines. Here, five strains of *Gambierdiscus* spp. were collected from Weizhou Island, a ciguatera hotspot in the Beibu Gulf, and identified by light and scanning electron microscopy and phylogenetic analyses based on large and small subunit rDNA sequences. Strains showed typical morphological characteristics of *Gambierdiscus caribaeus*, exhibiting a smooth thecal surface, rectangular-shaped 2′, almost symmetric 4″, and a large and broad posterior intercalary plate. They clustered in the phylogenetic tree with *G. caribaeus* from other locations. Therefore, these five strains belonged to *G. caribaeus*, a globally distributed *Gambierdiscus* species. Toxicity was determined through the mouse neuroblastoma assay and ranged from 0 to 5.40 fg CTX3C eq cell^−1^. The low level of toxicity of *G. caribaeus* in Weizhou Island, with CTX-contaminated fish above the regulatory level in the previous study, suggests that the long-term presence of low toxicity *G. caribaeus* might lead to the bioaccumulation of CTXs in fish, which can reach dangerous CTX levels. Alternatively, other highly-toxic, non-sampled strains could be present in these waters. This is the first report on toxic *Gambierdiscus* from the Beibu Gulf and Chinese waters and will provide a basis for further research determining effective strategies for ciguatera management in the area.

## 1. Introduction

Ciguatoxins (CTXs) are a group of polyether compounds mainly present in coral reef fish and rarely in invertebrates, which can cause ciguatera poisoning due to human consumption of CTX-contaminated seafood. CTXs are very potent and comprise one of the most significant marine biotoxin families, with 10,000–50,000 people affected worldwide annually [1]. CTX intoxication causes neurological abnormalities and gastrointestinal and cardiovascular dysfunction, with “paradoxical” sensory disturbance or “reversal” of temperature perception being the most characteristic symptoms [2]. In severe cases, intoxication symptoms can last for months or even years, and death is occasionally reported [3].

CTXs and its precursors are produced by epibenthic dinoflagellates of the genera *Gambierdiscus* and *Fukuyoa*. In the 1950s, Randall [4] proposed the famous food chain hypothesis, suggesting that CTXs originate from benthic microalgae in tropical seawater and are transferred to reef fish through the food chain. It was not until 1977 that the presence of *Diplopsalis* sp., the organism responsible for CTXs, was first confirmed in the Pacific Ocean on the Gambier Islands, French Polynesia, via a joint effort between Japan and France [5]. It was subsequently identified as a new genus and renamed *Gambierdiscus toxicus* [6]. Recently, globular *Gambierdiscus* was separated from anterior-posteriorly compressed *Gambierdiscus* based on morphology and defined independently as a new genus named *Fukuyoa* [7]. Unlike planktonic dinoflagellates that can produce blooms in surface waters, the genera *Gambierdiscus* and *Fukuyoa* mainly attach to macroalgae, dead corals, or rubble in benthic ecosystems exhibiting a patchy distribution and do not cause blooms visible to the naked eye.

Ciguatera, a human poisoning caused by the consumption of seafood [2], is predominantly endemic in tropical and subtropical Pacific, Atlantic, Caribbean, and Indian Ocean waters from 35° N to 37° S [8,9]. With increasing global warming and coastal disturbance caused by human activities, benthic dinoflagellates, including those of the genera *Gambierdiscus* and *Fukuyoa*, are experiencing geographic expansion or range shifts. As a result of expanding tourism and the global trade of reef fish, ciguatera incidents have increased significantly in recent years [10,11,12]. Owing to the difficulty in sampling benthic habitats, the study of CTXs and their toxin-producing organisms has been limited compared to that of planktonic microalgae [13,14]. This is particularly true in China, which has tropical and subtropical waters and has long been known to be a ciguatoxin-prone region [15,16,17] but with little history of CTX research.

As early as the 1970s, more than 20 species of carnivorous fish from the South China Sea islands, Guangdong coast, southern East China Sea, and Taiwan were reported to contain CTXs, although the toxicity was not determined [18]. In the 1980s and the 1990s, ciguatera incidents were first reported in Hong Kong and mainland China [19,20], but in 2007, CTX-contaminated fish were detected on the southern coast of China, including Weizhou Island and the Beibu Gulf [21]. Potential CTX-producing *Gambierdiscus* cells have been recorded in tropical and subtropical waters near the Xisha Islands, Hong Kong, Daya Bay, Hainan Island [22,23,24,25,26] and even in the temperate waters of Dayao Bay and Zhangzi Island in Dalian at 38°–39° N [27,28]. In addition, dormant cysts of the genus *Gambierdiscus* were found in Daya Bay and Dapeng Bay, Guangdong [29]. Recently, the potential CTX organisms *Fukuyoa ruetzleri* and *Fukuyoa* sp. HK Type 1 were recorded for the first time in the eastern waters of Hong Kong [30].

As reviewed by Chinain et al. [1], there are currently 18 species of *Gambierdiscus*, and the Pacific region has the highest species diversity and geographic distribution. Ciguatera poisoning has been widely reported in the central and southern Pacific, but is less frequently reported in the northern Pacific/Asia. In east and southeast Asia, the incidence of ciguatera is higher in coastal cities in southern China (Hong Kong, Foshan, Zhongshan) than in Japan (Okinawa Prefecture), whereas in other countries in the region, only isolated cases or small case series have been reported [17,31]. Unfortunately, fish exports from China have triggered ciguatera poisoning events in other countries; for example, between 2017–2019, Chinese exports of *Lutjanidae* led to at least two ciguatera outbreaks in Malaysia [1]. This is closely related to the fact that China, geographically a CTX-prone country, straddles tropical and subtropical sea areas, and has a diet that favors large banquets and large carnivorous fish [16]. However, it has been previously assumed that ciguatera events in China are caused by imported fish from Pacific island countries [32,33], and thus, research on Chinese ciguatoxic fish and potentially toxic dinoflagellates of the genera *Gambierdiscus* and *Fukuyoa* is limited. For decades, CTX-producing microalgae in Chinese waters have been named monotypic *Gambierdiscus toxicus* based on light microscopy [22,23,24,25,27,28], and precise species identification and toxicity determinations have not been conducted.

Until 2016, three precise *Gambierdiscus* species, *G. australes*, *G. caribaeus,* and *G. pacificus*, were first reported in China (Hainan Island) using light and scanning electron microscopy and phylogenetic trees based on D1–D3 and D8–D10 regions of the large subunit (LSU) rDNA [26]. This was the first accurate identification of *Gambierdiscus* in Chinese waters, but no toxicity measurements were conducted on these species. In 2018, Leung et al. [30] also identified *F.* *ruetzleri* and *Fukuyoa* sp. HK Type 1 for the first time in Chinese waters (Hong Kong) using similar morphological and molecular taxonomic methods and determined that *F. ruetzleri* and *Fukuyoa* sp. HK Type 1 were toxic and lethal to brine shrimp larvae using a bioassay methodology. Consequently, toxin characteristics of species-level well-defined *Gambierdiscus* in Chinese waters remain unknown to date. Given the presence of CTX-contaminated fish along the Chinese coast, there is an urgent need to conduct studies on species identification and toxicity, as well as on the physiological and ecological characteristics of the genera *Gambierdiscus* and *Fukuyoa*, in the region.

Weizhou Island (20°54′–21°10′ N, 109°00′–109°15′ E), located in the northwestern part of the South China Sea, is the largest island of the coast of the Guangxi province and in the Beibu Gulf, China. It is also the largest and youngest island formed by submarine volcanic eruptions in China and belongs to one of the most northerly distributed tropical coral reef ecosystems in the world [34,35]. In the past 20 years, coral reefs of Weizhou Island have continuously been degraded by human activities and extreme climates, such as the El Niño phenomenon in 1998 and extremely low temperatures in January–February, 2008 [36,37]. Corals that once covered the substrate are replaced by rubble, turf algae, an epilithic algae matrix, and macroalgae [36], all of which provide a suitable environment for the growth of epibenthic dinoflagellates, including CTX-producing *Gambierdiscus* and *Fukuyoa* and the transfer of CTXs along the food chain [38]. In 2007, researchers found CTX detection rates of 37.5% using the Cigua-Check^®^ kit and mouse bioassay in eight coral reef fish samples from Weizhou Island, and one *Epinephelus tauvina* sample among them exhibited the highest level of 135 ng P-CTX-1/kg meat, exceeding the safe threshold (100 ng P-CTX-1/kg meat) for consumption [21]. However, at that time, the origin and source of the CTXs in Weizhou Island were not studied.

In this study, five strains of *Gambierdiscus* spp. were isolated from dead coral and rubble on Weizhou Island in 2015–2017 in an effort to uncover the toxicity of ciguatoxin-producing microalgae in southern Chinese waters, to check whether *Gambierdiscus* produces CTXs and their potency, and to analyze their species composition and toxicity, highlighting a possible relationship with CTX-infected fish from Weizhou Island.

## 2. Results

### 2.1. Morphological Analysis

*Gambierdiscus* spp. belong to Dinophyta, Dinophyceae, Desmokontae, Gonyaulacales, Ostreopsidaceae, and *Gambierdiscus*. When observed under a light microscope, living cells were dark yellow and yellowish-brown, round or elliptical in the apical/antapical view, and lenticular in the lateral view. They had transverse and longitudinal grooves, and two flagella were found to arise from the cingulum and sulcus, respectively. The cells consisted of epithecae and hypothecae; however, they were mostly observed in the apical or antapical view under a light microscope. The average depth of living cells was 103.9 µm (76.1–132.6 μm) and the average width was 99.6 μm (70.8–123.8 μm).

**Figure 1:**
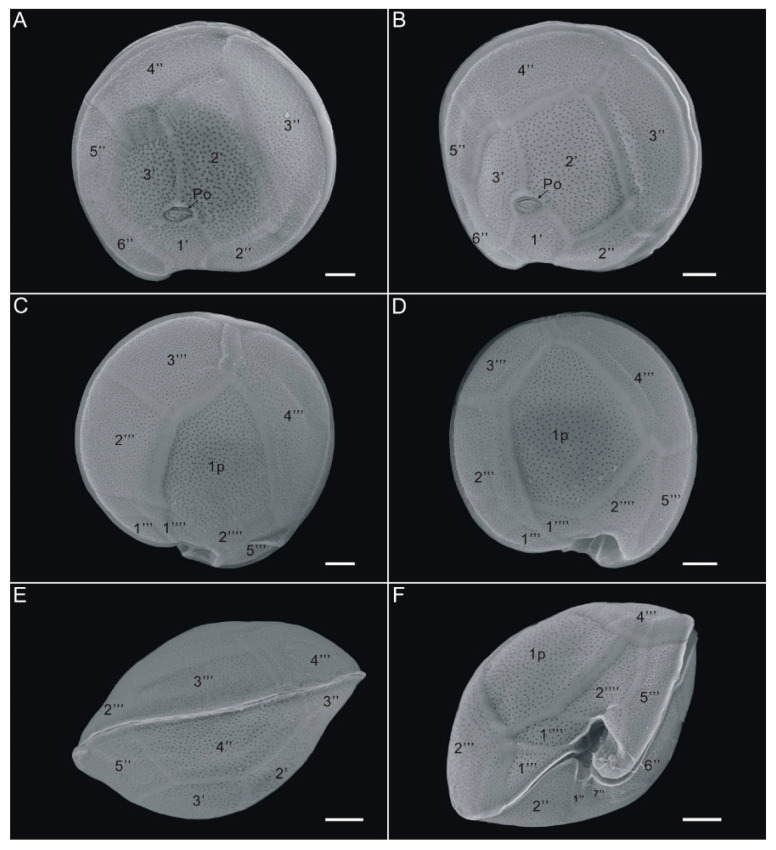
Scanning electron microscope images of *Gambierdiscus caribaeus* GCBG01 strain from Weizhou Island, Beibu Gulf of China. (**A**,**B**): apical view, (**C**,**D**): antapical view, (**E**): dorsal view, (**F**): ventral view. Scale bars: 10 µm.

**Figure 2:**
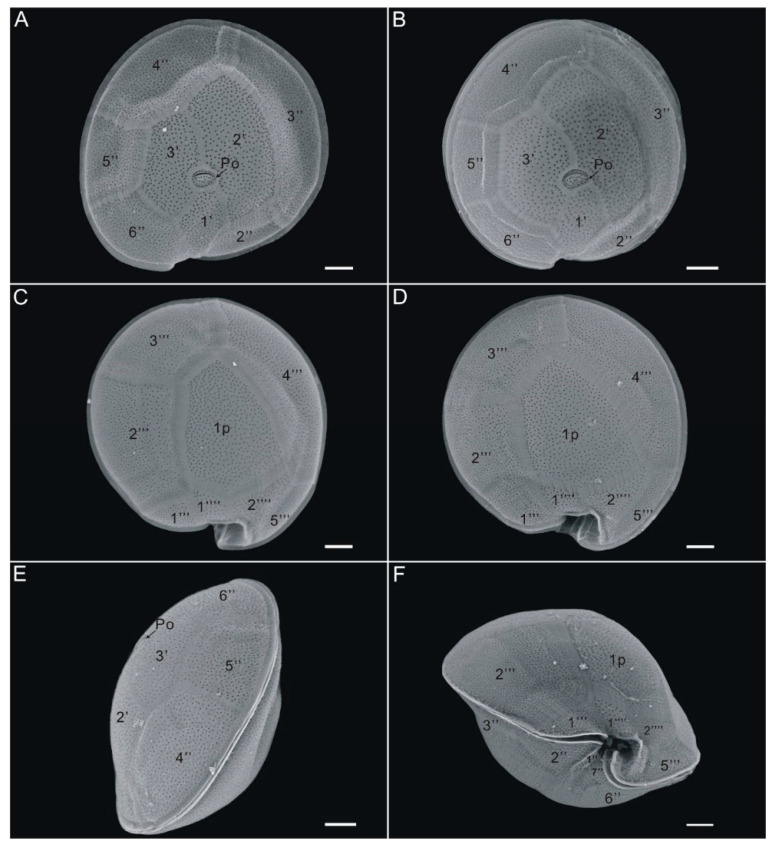
Scanning electron microscope images of *Gambierdiscus caribaeus* GCBG02 strain from Weizhou Island, Beibu Gulf of China. (**A**,**B**): apical view, (**C**,**D**): antapical view, (**E**): apical–lateral view, (**F**): ventral view. Scale bars: 10 µm.

**Figure 3:**
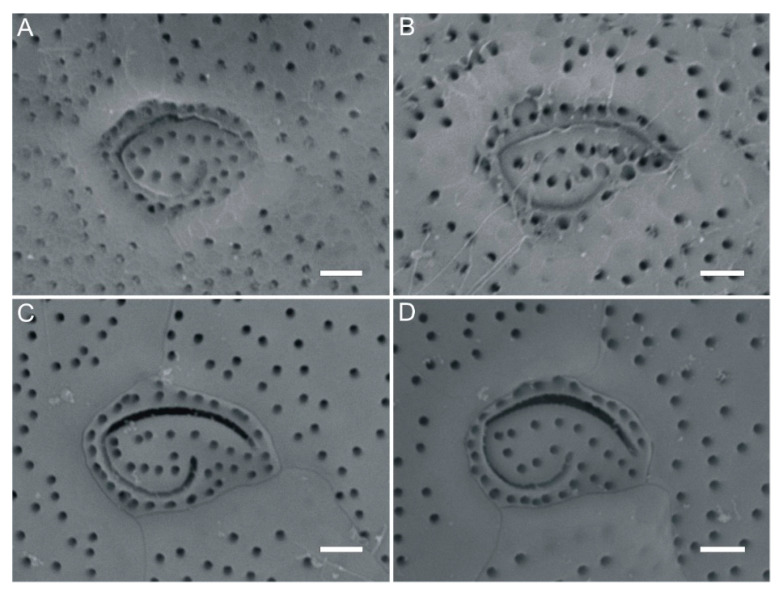
Scanning electron microscope images of fish-hook apical pore plates (Po) for *Gambierdiscus caribaeus* strains GCBG01 and GCBG02 from Weizhou Island, Beibu Gulf of China. (**A**,**B**): GCBG01, (**C**,**D**): GCBG02. Scale bars: 2 µm.

The toxic strain GCBG01 from Station S3 and the non-toxic GCBG02 from Station S4 were chosen as representative strains and observed by scanning electron microscopy. They were found to be nearly circular in both apical and antapical views, with a plate tabulation formula of Po, 3′, 7″, 5‴, 1p, and 2⁗ (cingular and sulcus plates were not measured; Figure 1 and Figure 2). The cell depth visualized through scanning electron microscopy averaged 77.0 μm (70.1–81.2 μm), and the cell width averaged 84.2 μm (73.4–93.6 μm). The cell surface was smooth and ornamented with many small, reticulated pores, and plates were separated by ridges. In the epithecae, an oval-shaped apical pore plate (Po) with a fishhook-like opening was observed near the center of the cell (Figure 1, Figure 2 and Figure 3), and three apical plates (′) were attached around Po, specifically 1′, 2′, and 3′, of which 1′ had a smaller area and was nearly pentagonally distributed on the side from the Po plate to the ventral edge, whereas 2′ had the largest area among the three apical plates and was rectangular. The precingular plates 2″, 3″, 4″, 5″, and 6″ could be clearly seen, with 3″ and 4″ being larger and occupying most of the left and dorsal sides of the cell, respectively, and 4″ being nearly symmetric (Figure 1 and Figure 2). No dorsal rostrum was found in either strain GCBG01 or GCBG02 (Figure 1 and Figure 2).

In the hypothecae, the postcingular plates 1‴, 2‴, 3‴, 4‴, and 5‴ could be clearly seen from the antapical view, with 4‴ and 2‴ being the largest (Figure 1 and Figure 2). The cell had a broad pentagonal posterior intercalary plate (1p) and two antapical plates 1⁗ and 2⁗. The 1p was connected to 2‴, 3‴, 4‴, 1⁗, and 2⁗ (Figure 1 and Figure 2). Among them, 1⁗ and 2⁗ were irregularly polygonal in shape and relatively small in area and were positioned close to the ventral side of the cell (Figure 1 and Figure 2). From the lateral view, the cingulum of the cell was narrow and slightly protruded in a beak-like shape, and at the ventral convergence, the cingulum met the sulcus to form a swirling deep groove (Figure 1E,F and Figure 2E,F). Thus, the morphological characteristics of strains GCBG01 and GCBG02 were consistent with those of *G. caribaeus*, which have been previously described [39,40,41,42,43,44].

### 2.2. Sequence Analysis

The average lengths of the D1–D3 LSU rDNA, D8–D10 LSU rDNA, and small subunit (SSU) rDNA sequences obtained from the five strains of *Gambierdiscus* from the Beibu Gulf were 1390, 908, and 1743 bp, respectively. Their sequence composition characteristics were similar to those of 50, 65, and 50 *Gambierdiscus* sequences that were used to construct a phylogenetic tree (Table 1). Among the three sequence types, the D8–D10 LSU rDNA sequence had the highest and lowest proportions of conserved (58.2%) and variable (40.4%) sites, respectively, which was similar to the composition of SSU rDNA sequences of conserved (57.4%) and variable (41.1%) sites but greater than that of the D1–D3 LSU rDNA sequences of conserved (30.4%) and variable (68.0%) sites.

### 2.3. Phylogenetic Tree Analysis

**Figure 4:**
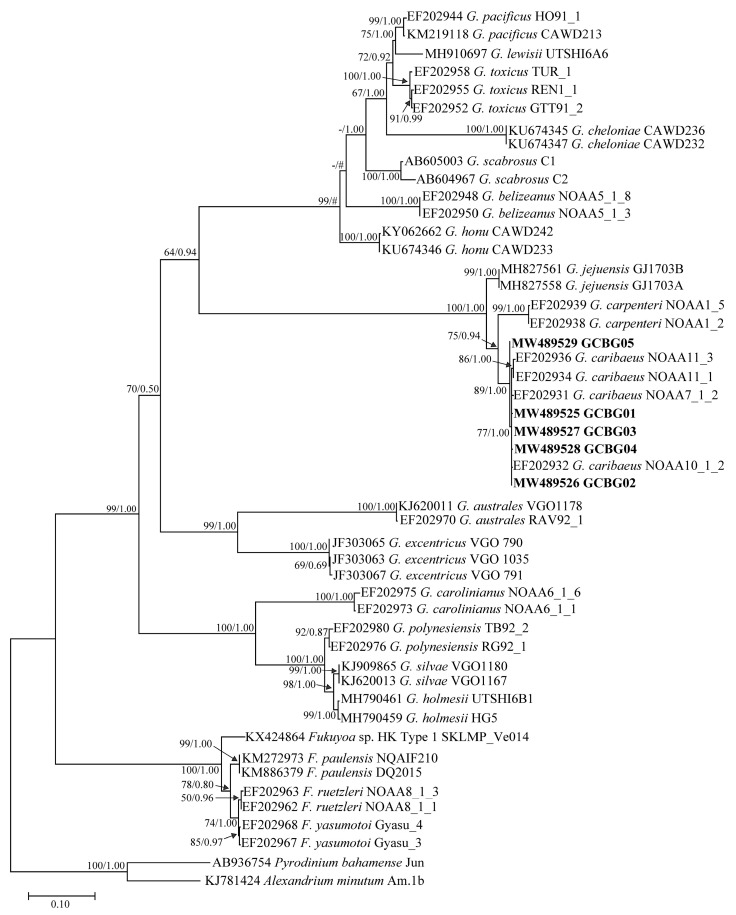
Phylogenetic tree constructed based on *Gambierdiscus* D1–D3 large subunit ribosomal DNA (D1–D3 LSU rDNA) sequences. Values at nodes indicate bootstrap values from the maximum likelihood method and posterior probabilities from the Bayesian inference method. Bootstrap values <50 and posterior probabilities <0.50 are not shown. # Indicates the topology; here, the maximum-likelihood tree differs from the Bayesian tree.

**Figure 5:**
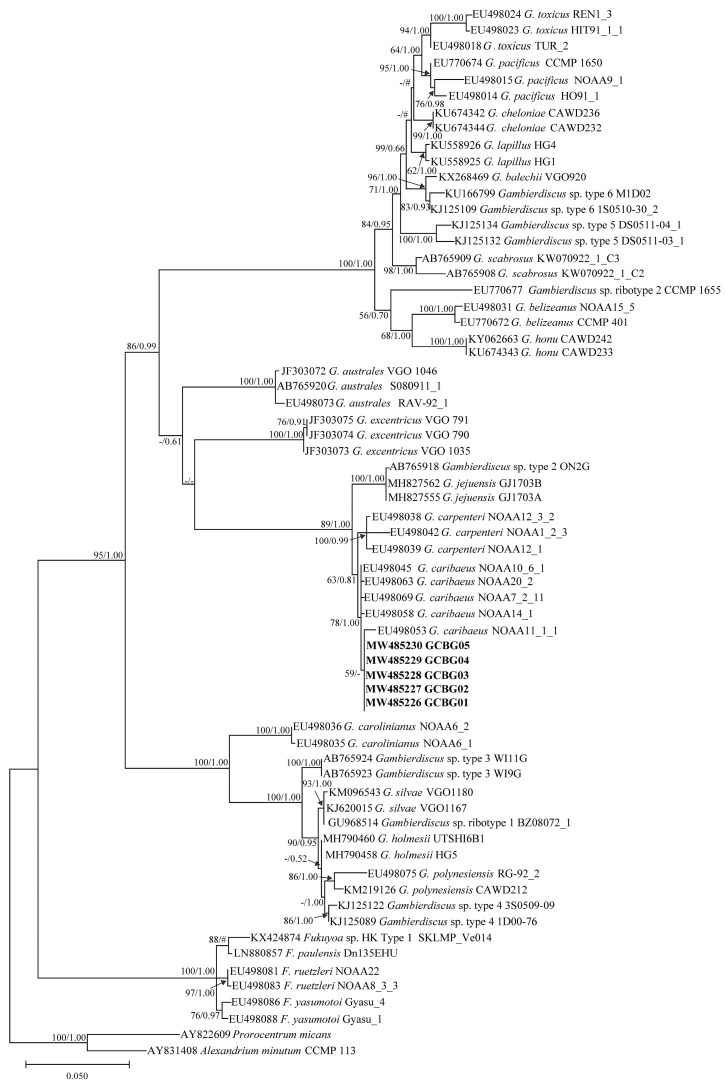
Phylogenetic tree constructed based on *Gambierdiscus* D8–D10 large subunit ribosomal DNA (D8–D10 LSU rDNA) sequences. Values at nodes indicate bootstrap values from the maximum likelihood method and posterior probabilities from the Bayesian inference method. Bootstrap values <50 and posterior probabilities <0.50 are not shown. # Indicates the topology; here, the maximum-likelihood tree differs from the Bayesian tree.

**Figure 6:**
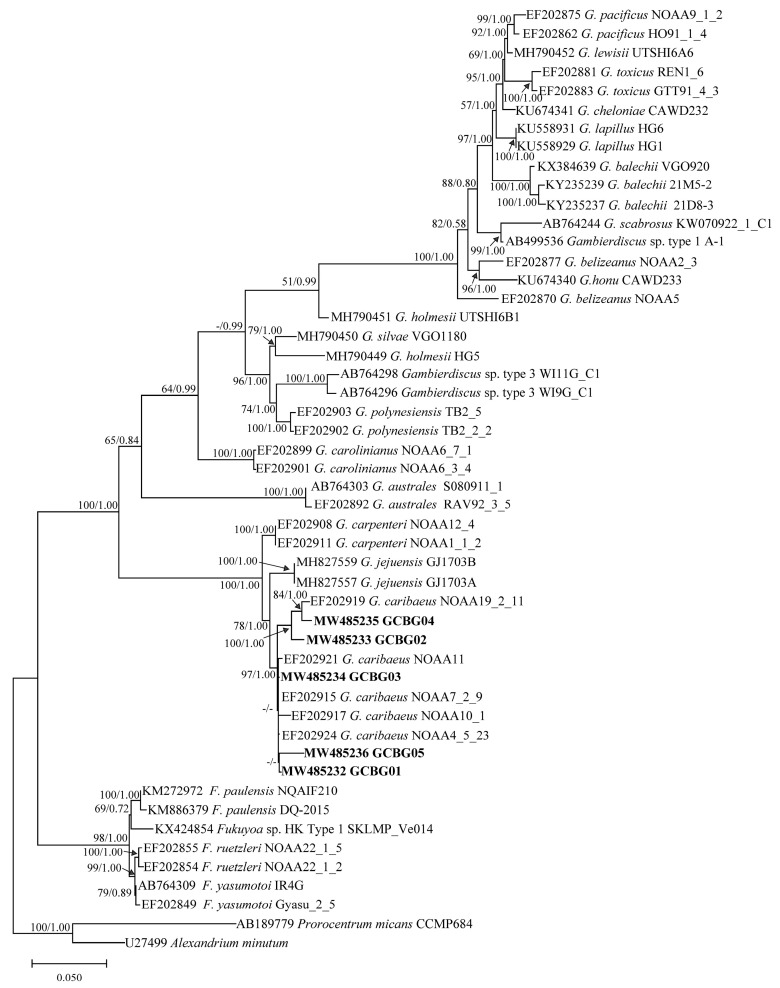
Phylogenetic tree constructed based on *Gambierdiscus* small subunit ribosomal DNA (SSU rDNA). Values at nodes indicate bootstrap values from the maximum likelihood method and posterior probabilities from the Bayesian inference method. Bootstrap values <50 and posterior probabilities <0.50 are not shown.

Phylogenetic trees of the D1–D3 LSU rDNA, D8–D10 LSU rDNA, and SSU rDNA were inferred using *Pyrodinium bahamense* and *Alexandrium minutum*, *Prorocentrum micans* and *A. minutum*, *P. micans* and *A. minutum*, respectively, as outgroups (Figure 4, Figure 5 and Figure 6). Sequences from 1–3 strains per species/ribotype were used for phylogenetic tree construction based on sequence availability. Except for *Gambierdiscus belizeanus* and *Gambierdiscus honu* on the D1–D3 LSU rDNA phylogenetic tree, *Gambierdiscus cheloniae* and *Gambierdiscus lapillus*, and *Fukuyoa paulensis* and *Fukuyoa* sp. HK Type 1 on the D8–D10 LSU rDNA phylogenetic tree, the topological structures of the trees using the maximum likelihood (ML) and Bayesian inference (BI) were largely consistent. Therefore, only ML trees are shown here.

Various species and genotypes in the genera *Fukuyoa* and *Gambierdiscus* were all independently branched and had good bootstrap and Bayesian posterior probability values, varying in 50–100/0.87–1.00, 62–100/0.93–1.00, and 79–100/0.89–1.00 for D1–D3 LSU rDNA, D8–D10 LSU rDNA, and SSU rDNA, respectively (Figure 4, Figure 5 and Figure 6). The five *Gambierdiscus* strains from Weizhou Island clustered with *G. caribaeus* from different marine areas in the world, exhibiting good support values of 89/0.94, 78/1.00, 94/0.98, respectively (Figure 4, Figure 5 and Figure 6). Combined with morphological characteristics described previously herein, these five sample strains were thus identified as *G. caribaeus*, a worldwide distributed species of Gambierdiscus. In agreement with the results of other studies [40,41], the result from our study highlighted that *G. caribaeus* constituted a clear large clade with *Gambierdiscus jejuensis* and *Gambierdiscus carpenteri* in support of 100/1.00, 89/1.00, 100/1.00 values. On D1–D3 and D8–D10 LSU rDNA trees, *G. jejuensis* was a basal species, whereas *G. caribaeus* and *G. carpenteri* formed a sister branch (Figure 4 and Figure 5). In SSU rDNA tree, *G. carpenteri* was a basal species and *G. caribaeus* and *G. jejuensis* formed a sister branch (Figure 6).

Genetic *distances* of D1–D3 LSU rDNA, D8–D10 LSU rDNA, and SSU rDNA sequences were calculated using the *p* distance (uncorrected genetic distance). A detailed comparison of genetic distances is shown in Table S1. In brief, the intraspecific genetic distances of D1–D3 LSU rDNA, D8–D10 LSU rDNA, and SSU rDNA sequences for *G. caribaeus* (including Weizhou Island strains) were 0.010 ± 0.005 (0.002–0.022), 0.008 ± 0.004 (0.000–0.013), and 0.014 ± 0.009 (0.001–0.035), respectively, and they were significantly smaller than the interspecific distances between *G. caribaeus* and other *Gambierdiscus* species, that is, 0.334 ± 0.109 (0.052–0.418), 0.155 ± 0.058 (0.016–0.234), and 0.143 ± 0.045 (0.017–0.187), respectively. The genetic distance data confirmed that the five *Gambierdiscus* strains from Weizhou Island, Beibu Gulf belonged to *G. caribaeus*.

### 2.4. Toxin Analysis

Among the five strains of *G.* *caribaeus* tested, only GCBG01 isolated from the northeast side of Weizhou Island (S3) produced CTX-like toxicity with a low value of 0.54 fg CTX3C eq cell^−1^, whereas no toxicity was detected in the remaining four strains (Table 2).

## 3. Discussion

To date, eighteen species and five ribotypes have been described within the genus *Gambierdiscus*. It is not surprising to find *G. caribaeus* in Weizhou Island, as this species is one of the most widely distributed *Gambierdiscus* species [45], recorded in many waters, including the Caribbean, North Atlantic, and Pacific (Figure S1). Moreover, it has also been found in the waters of Hainan Island, China, which is adjacent to the Weizhou Island [26].

All available *G. caribaeus* D1–D3 LSU rDNA and D8–D10 LSU rDNA sequences in the National Center for Biotechnology Information (NCBI) database were downloaded, and the genetic distance matrix was calculated through MEGA-X v10.1.8 and analyzed with principal coordinate analysis (PCoA) in RStudio (Figure S2). In the D1–D3 LSU rDNA PCoA plot, although *G. caribaeus* strains GCBG02-05, HF2, and 64B3 seemed to be separated, the explanatory degree of the X-axis and Y-axis together was only 34.16% of the total variation, whereas in the D8–D10 LSU rDNA PCoA plot, the explanatory degree of the X-axis and Y-axis together was 10.03% of the total variation (Figure S2). Therefore, sequence differences between different geographical sources of *G. caribaeus* were very small, and there was no obvious clustering pattern based on geographical location. Referring to D8–D10 LSU rDNA for *G. caribaeus*, which had more sequences than D1–D3 rDNA and SSU rDNA sequences in NCBI, the genetic distance for D8–D10 LSU rDNA among the five strains of *G. caribaeus* in this study and strains NOAA11_1_1 from Carrie Bow Cay, Belize (Caribbean), NOAA20_5 from Grand Cayman Island (Caribbean), and TF9G and TF26G from Koh Wai, Thailand was closest, with a value of 0.016 ± 0.006 (0.001–0.024).

In this study, five strains of *G. caribaeus* were isolated from Weizhou Island, and only one strain (GCBG01) had detectable toxicity at 0.54 fg P-CTX-1 eq cell^−1^. This represents the first report of toxic *Gambierdiscus* not only from Beibu Gulf, but also from Chinese waters. The toxicity magnitude of *G. caribaeus* strains from Weizhou falls within the *G. caribaeus* toxicity range (i.e., <the limit of detection to 903.70 fg CTX3C eq. cell^−1^) has been reported in previous studies (Table 3). *G. caribaeus* has been thought to be a low and slow toxin-producing species [44,46,47]; to date, all documented toxicity for this species from the Caribbean and Pacific regions is less than 5.40 fg P-CTX-1 eq cell^−1^ (Table 3). However, several other strains recently isolated from the Canary Islands exhibit surprisingly high toxicity; in particular, strain VGO1367 shows an extremely high CTX-like toxicity of 903.70 fg CTX3C eq. cell^−1^, which is comparable to that in the most highly toxic species, including *Gambierdiscus excentricus* in the Atlantic and *Gambierdiscus polynesiensis* in the Pacific [1,46,48]. In this regard, it should be attributed to the association between the toxicity and the geographical origin of the strain. Rossignoli et al. [47] have suggested that some species in the genus *Gambierdiscus*, like *G. australes* and *G. silvae*, have a similar CTX-like toxicity for strains from different locations, but *G. caribaeus* and *G. excentricus* differ considerably due to the origin of the strain. The toxicity of *G. caribaeus* from Weizhou Island here seemed to reinforce the fact that the *G. caribaeus* in the Pacific Ocean has low toxicity. The mechanism underlying for the toxicity of some *Gambierdiscus* species is unrelated to the geographical origin, whereas other species is related. Therefore, this uncertainty remains a challenge that needs to be unraveled.

Production of CTX or CTX-like compounds is more dependent on intrinsic genetic factors (species/strain) than on extrinsic environmental conditions (temperature, pH, light, salinity, nutrients, and growth stage) [9,45,55,56]. In the Caribbean and Gulf of Mexico, a 1740-fold interspecies variations in *Gambierdiscus* and *Fukuyoa* toxicity were observed [46]. Here, for *G. caribaeus*, the most toxic strainVGO1367 from Canary Islands was 4755-fold more toxic than the less toxic strain Keys Jar 7 from Florida Keys (Table 3). In comparison, combined effects of light, salinity and temperature on *Gambierdiscus* culture can lead to a maximum 200-fold difference in toxicity [57]. It has also been reported that *Gambierdiscus* spp. under extreme conditions such as low temperature/increased depth, low light, and stationary and decline phase produce more CTXs than under optimal growth conditions and in the exponential phase. Therefore, if the optimal batch cultivation conditions are changed, the CTX-like toxicity of *G. caribaeus* strains in this study might increase, and it could also be detected in GCBG02–GCBG05 strains currently below the detection limit. Unfortunately, this study focused on *Gambierdiscus* species identification and preliminary toxicity analysis in Weizhou Island and did not test their toxicity associated with different growth phases and growth conditions, which to some extent, limits the comprehensive understanding of the toxin-producing characteristics of *G. caribaeus* and ciguatoxic fish in the region.

The non-toxic and low-level toxicity of *G. caribaeus* in this study seems to contradict the 37.5% CTX detection rate and 12.5% CTX exceedance rate of Weizhou Island coral reef fish. In addition to the environmental factors mentioned previously herein that might lead to a higher CTXs production in natural water bodies compared to that in the current strains GCBG01–GCBG05, the following reasons are also possible:

(1) Collection dates for fish samples in the study of Xu et al. [21] and those of CTX-producing microalgae collection in this study were different. As a result, *Gambierdiscus* species and their corresponding toxicity might differ between the two time periods. Further, even at the same sampling time, asynchrony of *Gambierdiscus* spp. abundance and toxicity have been documented in both the Pacific and Caribbean regions [56,58].

(2) Additionally, other more toxic *Gambierdiscus*/*Fukuyoa* species are present on Weizhou Island but have not been recognized because of their low abundance. Scientists have recently identified *G. pacificus*, *G. australes*, and *G. caribaeus* in the Hainan Island [26] and *F.* *ruetzleri* in Hong Kong waters [30], not far from Weizhou Island. According to other studies, all of these species are generally more toxic than *G. caribaeus* [44,52,59]. If the sampling intensity and frequency are enhanced in Weizhou Island, other *Gambierdiscus*/*Fukuyoa* spp. with medium- or high-level toxicity, such as *G. polynesiensis*, which has proven to be the most toxic and major toxin producer in the Pacific Ocean with a wide geographic distribution, are likely to be found [1]. The average toxicity of *G. polynesiensis* from French Polynesia calculated by Longo et al. [48] is 3285 fg CTX3C eq cell^−1^, a 75.8-fold increase compared to the average *G. caribaeus* toxicity, specifically 43.33 ± 192.25 fg CTX3C eq cell^−1^ (Table 3).

(3) It cannot be ruled out that it was indeed the low toxicity of *G. caribaeus* that resulted in ciguatoxic fish in Weizhou Island, because this species has a wider range of tolerance to environmental conditions of temperature, salinity, and light than most other species of the genus *Gambierdiscus*, consistent with their broad geographic distribution [9,50,60]. The long-term presence of *G. caribaeus* with low toxicity in the water column will result in bioaccumulation in fish and will lead to dangerous CTX levels. Litaker et al. [46] have analyzed 33 strains representing seven *Gambierdiscus* and one *Fukuyoa* species from the Caribbean and Gulf of Mexico and have found that all except *G. excentricus* can induce high CTX-like toxicity, at 469 fg CTX3C eq cell^−1^, comparable with the highly toxic isolates from the Pacific [1,61]. All other six species were low toxin-producers, varying from 0.27–19.6 fg CTX3C eq cell^−1^ [46], but that does not change the fact that the Caribbean and Gulf of Mexico remain among the most CTX-prone areas in the world. Our speculation needs to be verified through the long-term tracking of the abundance and species composition of *Gambierdiscus*/*Fukuyoa* spp. in Weizhou Island in the future.

## 4. Conclusions

In this study, species identification and toxicity analyses of CTX-producing microalgae in Weizhou Island were conducted for the first time, and five strains of *G. caribaeus* were identified based on thecal morphology and a molecular characterization. The toxicity was determined to be 0–5.40 fg CTX3C eq cell^−1^, which represents the first report of toxic *Gambierdiscus* in Chinese waters. Further studies are strongly recommended to better explain the correlation between the causative organism abundance, species composition, species/strain-specific toxicity, and the risk of CTX-contaminated fish in Weizhou Island waters and to develop better strategies for ciguatera management in China.

## 5. Materials and Methods

### 5.1. Study Area and Sample Collection

**Figure 7:**
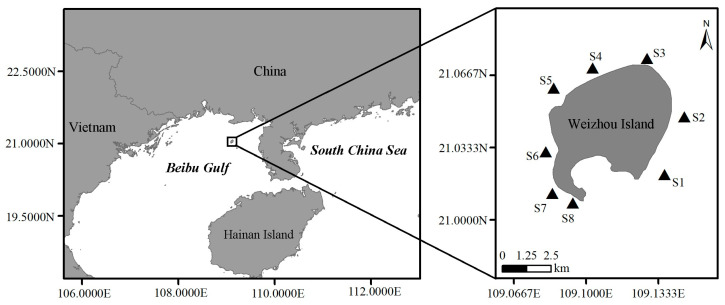
Sampling sites.

Eight sites were sampled on Weizhou Island in October–2014–2016 (Figure 7). Benthic macroalgae and hard dead coral reef rubble were collected by diving. Cells of benthic dinoflagellates including *Gambierdiscus* attached to the macroalgae and hard dead coral were released by vigorous shaking, and 20–200 μm particles were collected through filtration for further cell isolation and monoclonal strain establishment, adding the 20 μm-filtered seawater and 1 mL of reduced enriched K medium (removing Si and Tris) [62] to the samples to keep *Gambierdiscus* cells alive but prevent fast reproduction.

### 5.2. Culture Establishment

Field samples were transported to the laboratory and observed using an inverted microscope (TS100, Nikon, Tokyo, Japan). The cell size and shape of *Gambierdiscus* sp. were observed and photographed (NIS-Elements D 4.50.00), and these were then isolated with a capillary tube under an inverted microscope. The cells were aspirated and placed onto a glass plate with K medium, washed repeatedly 3–4 times, and then single cells were cultured in a 96-well plate in a light incubator (GHP-9270N, Ningbo Laifu Technology Co., Ningbo, China) at 23 °C, 150 μE·m^–2^ s^–1^, with a 14 h:10 h light:dark cycle. After 4–6 weeks of culture, well-grown cells were selected and transferred to a 24-well plate for further growth, and 2–3 weeks later, cells were transferred to cell culture flasks (25 cm^2^, Fisher Scientific Co., Waltham, MA, USA) and maintained as a monostrain culture of *Gambierdiscus* sp. (Table S2).

### 5.3. Scanning Electron Microscopy

Glutaraldehyde solution was added to the *Gambierdiscus* culture growing in exponential growth phase for 35 h for cell fixation, at a final solution concentration of 2.5%. Fixed samples (5 mL) were filtered and collected through a 3 μm polycarbonate membrane (Merck Millipore Ltd., Burlington, MA, USA) and then desalted in a salinity gradient. Using sterile seawater at a concentration of 100%, desalination was performed with a concentration gradient of 90%, 70%, 50%, 30%, 0%, and 0% (ultrapure water), with each iteration lasting for 30 min. The filter membrane was then dewatered in different concentrations of ethanol with a concentration gradient of 30%, 50%, 70%, 90%, 100%, and 100%, with each iteration conducted for 30 min, dried with a critical point drier (Joel Hi-Tech, Dalian Co., Dalian, China), and gold-coated in a sputter coater. Lastly, the *Gambierdiscus* cells were observed and photographed under a scanning electron microscope (TM-1000 Tabletop Microscope, Hitachi High-Technologies Corporation, Tokyo, Japan). The plate formula for the morphology description was the same as that described in previous publications [39,63,64].

### 5.4. DNA Extraction and PCR Amplification

DNA was extracted from *Gambierdiscus* cells growing in exponential phase using the BioFastSpin Plant Genomic DNA Extraction Kits (Bioer, Hangzhou, China) following the manufacturer’s instructions. DNA extraction was confirmed through electrophoresis. Characteristic D1–D3 LSU rDNA, D8–D10 LSU rDNA, and SSU rDNA fragments were amplified through PCR using the primers listed in Table S3. The PCR reaction included 20 µL 2 × Es Taq Master Mix (CoWin Biosciences, Cambridge, MA, USA), forward and reverse primers (1 µL each), DNA template (1 µL), and ddH_2_O (17 µL). The PCR program was run on a Biometra EasyCycler Gradient, and comprised pre-denaturation at 94 °C for 5 min, 35 cycles of 94 °C for 30 s, 56 °C for 30 s, and 72 °C for 30 s, and elongation at 72 °C for 5 min. The D1–D3 LSU rDNA and D8–D10 LSU rDNA PCR products were directly Sanger sequenced at the TsingKe Biological Technology Company (Beijing, China).

Direct SSU rDNA Sanger sequencing failed, and these samples were further cloned before sequencing. Therefore, SSU rDNA was extracted from PCR agarose gels, purified using the Universal DNA Purification Kit (Tiangen, DP214, Beijing, China), and then ligated with the pMD19-T vector overnight at 16 °C. Receptor cells (*Escherichia coli* DH5α) were removed from the −80 °C storage freezer and thawed on ice. DH5α was added to the ligation product, mixed well, incubated in an ice bath for 30 min, and heat shocked at 42 °C for 60–90 s, which was followed by rapid transfer to an ice bath for 2–3 min. Then, 500 µL of Luria-Bertani medium (without antibiotics) was added to the mixture, mixed well, and incubated at 37 °C for 45 min (150 rpm) in a shaker. The mixture (200 µL) was pipetted into a Luria-Bertani plate (containing ampicillin, Isopropyl β-d-1-thiogalactopyranoside, and X-Gal) and evenly spread. The plate was incubated a 37 °C overnight. Single colonies were picked for PCR confirmation, and the positive ones were chosen for Sanger sequencing. All sequences were deposited in GenBank (Table S2).

### 5.5. Phylogeny and Sequence Analysis

The D1–D3 LSU rDNA, D8–D10 LSU rDNA, and SSU rDNA sequences of the five *Gambierdiscus* isolates (GCBG01, GCBG02, GCBG03, GCBG04, and GCBG05) were searched in the NCBI website (http://www.ncbi.nih.gov) using BLAST, and sequences related to these strains with high similarity and coverage were downloaded. Totals of 44, 60, and 44 sequences were obtained, respectively. Sequences were subjected to multiple sequence alignment using ClustalW in MEGA-X v10.1.8, and redundant bases were sorted and truncated to 1035 bp, 914 bp, and 1788 bp for the D1–D3 LSU rDNA, D8–D10 LSU rDNA, and SSU rDNA, respectively. Base composition, conserved sites, variant sites, parsimony informative sites, monomorphic sites, and conversion/inversion ratios were also calculated using MEGA-X v10.1.8. The substitution model and rates among sites were determined by running the software Modeltest 3.7, showing the best selection as GTR + G, which was used in the construction of the ML and BI trees.

ML trees were constructed using software MEGA-X v10.1.8, with 1000 bootstrap replications, partial deletion of gaps/missing data treatment, and a site coverage 95% cutoff. BI trees were inferred using the Bayesian algorithm in MrBayes v3.1.2, and 1 × 10^6^ runs of three hot chains and one cold chain were performed independently under four Markov chains, with sampling performed every 500 generations. The analysis was thought to reach stability, that is, a standard error <0.01, when the program ended. Phylogenetic trees were viewed with the software FigTree v1.4.0 and saved in PDF format. The GenBank accession number, species and strain names, bootstrap values, and posterior probability values were added to the major branch node using Adobe Acrobat Pro v11.0.5.

### 5.6. CTX-Like Toxicity Analysis

The 10^6^–10^7^ *Gambierdiscus* cells in the early stationary phase were collected by centrifugation at 2000× *g* at 4 °C and stored at −20 °C by adding methanol. CTX-like toxins were released from cells using a sonicator probe (Branson Digital Sonifier, Branson Ultrosonics Corporation, Brookfield, CT, USA) for 5 s on, 1 s off, and 80% amp. After sonication, a drop of each extract was placed on a microscope slide to verify cell disruption. The primary extract was centrifuged at 2000× *g* for 5 min at 4 °C, and the supernatant was collected. Residual cells were extracted for 2 min using a vortex mixer (Vortex-6, Qilinbeier, Jiangsu, China), and the supernatant was combined with the primary extracted supernatant. CTX-like toxins in the supernatant were then extracted using a 3:2:2 volume ratio of methanol:MilliQ water:dichloromethane and dried in a rotary evaporator; then, methanol was added, and the extract was stored at −20 °C for final toxicity measurements. Toxicity was determined using mouse neuroblastoma (Neuro-2a cells; ATCC, CCL131; ATCC, Manassas, VA, USA) according to the mouse neuroblastoma assay described by Xu et al. [64].

## Figures and Tables

**Table 1:** Sequence composition used in *Gambierdiscus* phylogenetic tree.

Gene	Analysis Length	Average Content (%)				Conserved Site	Variable Site	Parsimonious Information Site	Monomorphic Site	Conversion/Transversion Ratio
		A	T	G	C					
D1–D3 LSU rDNA	1035	25.3	25.1	30.8	18.8	323	696	661	35	0.9
D8–D10 LSU rDNA	914	26.1	25.8	27.8	20.3	532	369	281	87	1.5
SSU rDNA	1774	24.9	27.1	28.1	19.9	1016	728	578	149	1.6

**Table 2:** Ciguatoxin (CTX)-like toxicity of *Gambierdiscus caribaeus* isolates from Weizhou Island, Beibu Gulf of China (fg CTX3C eq cell^−1^).

Strains	Species	Toxicity
GCBG01	*G. caribaeus*	5.4
GCBG02	*G. caribaeus*	ND
GCBG03	*G. caribaeus*	ND
GCBG04	*G. caribaeus*	ND
GCBG05	*G. caribaeus*	ND

ND: Toxicity not detected.

**Table 3:** Summary on *Gambierdiscus caribaeus* ciguatoxin (CTX)-like toxicity in different strains.

Strain	Locality	Toxicity	Methodology	References
NOAA 7 (CCMP 1652)	Mataiva, Tahiti, South Pacific	2,3-dihydroxy CTX3C *	LC-MS/MS	[49]
NOAA 20 (CCMP 1651)	Grand Cayman Island, Caribbean	2,3-dihydroxy CTX3C *	LC-MS/MS	[49]
CCMP 401	St. Barthelmy Island, Caribbean	2,3-dihydroxy CTX3C *	LC-MS/MS	[49]
CCMP 1733	Carry Bow Cay, Belize, Caribbean	2,3-dihydroxy CTX3C *	LC-MS/MS	[49]
TF9G	Koh Wai, Trat, Thailand	≥100 × 10^−4^ MU/1000 cells	MBA	[43,50]
Gam 19	Carrie Bowe Caye Belize, Caribbean	<LOD	CBA	[51]
Pat HI Jar 2 Gam 2	Big Island, Hawaii, USA, Pacific	<LOD	CBA	[51]
CCMP1733	Carrie Bow Cay, Belize, Caribbean	0.80 ± 0.43 fg CTX3C eq cell^−1^	CBA	[46]
CCMP 1651	Grand Cayman Island, Caribbean	0.48 ± 0.04 fg CTX3C eq cell^−1^	CBA	[46]
SW gam 5	Southwater Cay, Belize, Caribbean	1.52 ± 0.26 fg CTX3C eq cell^−1^	CBA	[46]
CBC gam1	Carrie Bow Cay, Belize, Caribbean	0.62 ± 0.12 fg CTX3C eq cell^−1^	CBA	[46]
Mexico Algae 1	Cancun, Mexico	1.29 ± 0.40 fg CTX3C eq cell^−1^	CBA	[46]
Dive 1 FA	Carrie Bow Cay, Belize, Caribbean	0.69 ± 0.19 fg CTX3C eq cell^−1^	CBA	[46]
Keys Jar 7	Florida Keys, USA	0.19 ± 0.03 fg CTX3C eq cell^−1^	CBA	[46]
Bill Hi Gam8	Waikiki Beach, Honolulu, Hawaii	1.60 ± 1.00 fg CTX3C eq cell^−1^	CBA	[52]
CUB4A5	Cienfuegos coast, south-central Cuba	<LOD	RBA	[53]
VGO1362	La Gomera Porto-Playa Santiago, Canary Islands	6.00 fg CTX3C eq cell^−1^	CBA	[47]
VGO1364	La Gomera Porto-Playa Santiago, Canary Islands	25.9 ± 5.0 fg CTX3C eq cell^−1^	CBA	[47]
VGO1365	La Gomera Porto-Playa Santiago, Canary Islands	<LOD	CBA	[47]
VGO1366	La Gomera Porto-Playa Santiago, Canary Islands	5.00 fg CTX3C eq cell^−1^	CBA	[47]
VGO1367	La Gomera, San Sebastián-Playa la Cueva, Canary Islands	903.7 ± 158.9 fg CTX3C eq cell^−1^	CBA	[47]
IRTA-SMM-17-03	Tamaduste, El Hierro, Canary Islands	<LOD	CBA	[44]
IRTA-SMM-17_03	Tamaduste, El Hierro, Canary Islands	0.13–0.21 fg 51-hydroxy CTX3C eq cell^−1^	Immunoassay	[54]
IRTA-SMM-17_03	Tamaduste, El Hierro, Canary Islands	1.3–2.4 fg CTX3C eq cell^−1^	Immunoassay	[54]
GCBG01	Weizhou Island, Beibu Gulf, China	5.40 fg CTX3C eq cell^−1^	CBA	This study
GCBG02	Weizhou Island, Beibu Gulf, China	<LOD	CBA	This study
GCBG03	Weizhou Island, Beibu Gulf, China	<LOD	CBA	This study
GCBG04	Weizhou Island, Beibu Gulf, China	<LOD	CBA	This study
GCBG05	Weizhou Island, Beibu Gulf, China	<LOD	CBA	This study

*, Qualitative results; LOD, limit of detection; MBA, mouse bioassay; RBA, receptor binding assay; CBA, cell-based assay; the toxicity of VGO1362 and VGO1366 was roughly determined from Figure 3 in [47].

## Data Availability

Data are available upon request, please contact the first author Yixiao Xu.

## Supplementary Materials

The following is available online at https://www.mdpi.com/article/10.3390/toxins13090643/s1, Figure S1: global distribution map of *Gambierdiscus caribaeus*, Figure S2: principal coordinate analysis (PCoA) based on the *p* distance matrices of *Gambierdiscus caribaeus* D1–D3 LSU rDNA and D8–D10 LSU rDNA sequences downloaded from the NCBI database, Table S1: the *p* distance (uncorrected genetic distance) comparison of D1–D3 LSU rDNA, D8–D10 LSU rDNA, and SSU rDNA sequences, Table S2: sample information, Table S3: primers and PCR product information.
