# Supplementary material for: Ciguatoxin-Producing Dinoflagellate Gambierdiscus in the Beibu Gulf: First Report of Toxic Gambierdiscus in Chinese Waters
Source: Toxins (Basel). 2021 Sep 10;13(9):643. doi: 10.3390/toxins13090643 (PMC8473099; doi:10.3390/toxins13090643)
Supplement: Supplementary file 1 [file toxins-13-00643-s001.zip › toxins-1338519-supplementary (1).pdf]

toxins

Supplementary Materials: Ciguatoxin-Producing Dinoflagellate *Gambierdiscus* in the Beibu Gulf: First Report of Toxic *Gambierdiscus* in Chinese Waters

Yixiao Xu, Xilin He, Wai Hin Lee, Leo Lai Chan, Douding Lu, Pengbin Wang, Xiaoping Tao, Huiling Li and Kefu Yu

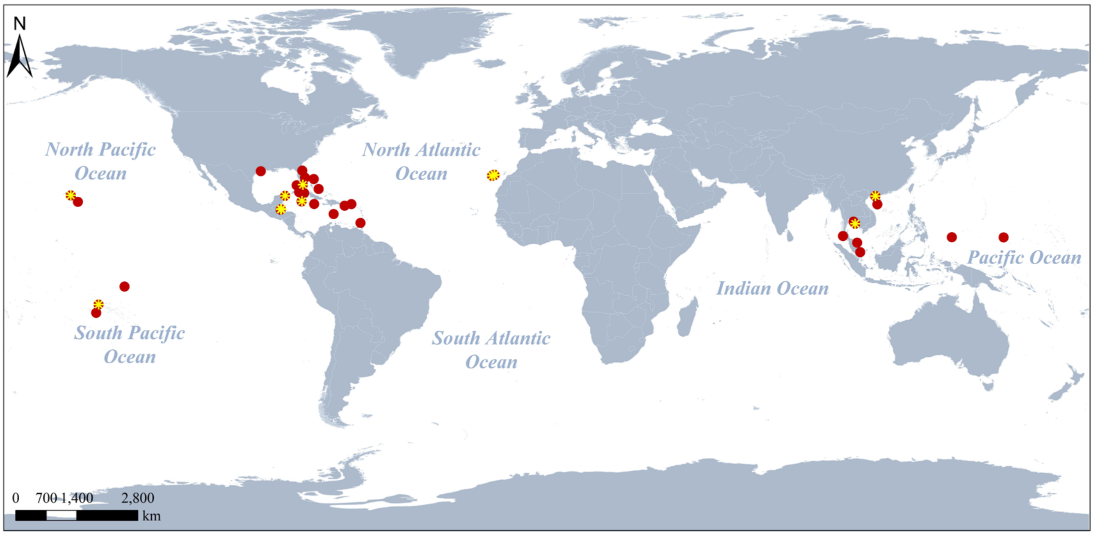

Figure S1. Global distribution map of *Gambierdiscus caribaeus*. Red dots indicate the presence of *G. caribaeus*, and yellow asterisks indicate the presence of *G. caribaeus* containing ciguatoxin (CTX)-like toxicity.

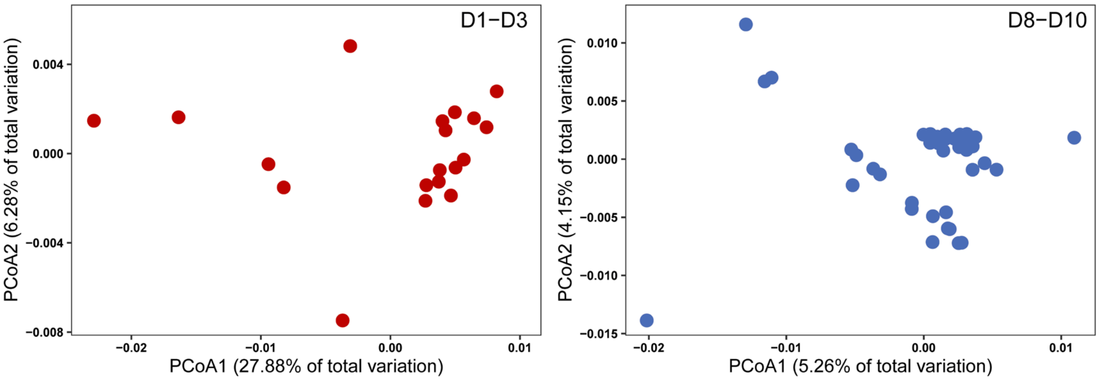

Figure S2. Principal coordinate analysis (PCoA) based on the *p* distance matrices of *Gambierdiscus caribaeus* D1–D3 LSU rDNA and D8–D10 LSU rDNA sequences downloaded from the NCBI database.

Table S1. The *p* distance (uncorrected genetic distance) comparison of *Gambierdiscus* D1–D3 LSU rDNA, D8–D10 LSU rDNA, and SSU rDNA sequences.

	G. caribaeus (WZ)	*G. caribaeus* (WZ)-*G. caribaeus* (Tree)	*G. caribaeus* (WZ)-*G. carpenteri* (Tree)	*G. caribaeus* (WZ)-*G. jejuensis* (Tree)
D1–D3 LSU	0.007 ± 0.002(0.002–0.010) [a]	0.013 ± 0.005 (0.007–0.022) [b]	0.069 ± 0.005 (0.064–0.077) [c]	0.060 ± 0.006 (0.052–0.068) [d]
D8–D10 LSU	0.006 ± 0.005 (0.000–0.011) [e]	0.010 ± 0.003 (0.005–0.013) [f]	0.022 ± 0.005 (0.016–0.031) [g]	0.029 ± 0.005 (0.022–0.039) [h]
SSU	0.019 ± 0.009 (0.002–0.035) [i]	0.014 ± 0.009 (0.001–0.032) [j]	0.028 ± 0.008 (0.018–0.035) [k]	0.031 ± 0.007 (0.022–0.038) [l]

WZ: Weizhou Island strains; Tree: strains included in Figure 4-6. [a] MW489525–MW489529. [b] MW489525–MW489529, EF202931, EF202932, EF202934, and EF202936. [c] MW489525–MW489529, EF202938, and EF202939. [d] MW489525–MW489529, MH827558, and MH827561. [e] MW485226–MW485230. [f] MW485226–MW485230, EU498045, EU498053, EU498058, EU498063, and EU498069. [g] MW485226–MW485230, EU498038, EU498039, and EU498042. [h] MW485226–MW485230, MH827555, and MH827562. [i] MW485232–MW485236. [j] MW485232–MW485236, EF202915, EF202917, EF202919, EF202921, and EF202924. [k] MW485232–MW485236, EF202908, and EF202911. [l] MW485232–MW485236, MH827557, and MH827559.

Table S2. Sample information.

Strains	Location	GenBank Accession No.		
		D1-D3 LSU rDNA	D8-D10 LSU rDNA	SSU rDNA
GCBG01	S3 (109.1301E, 21.0772N)	MW489525	MW485226	MW485232
GCBG02	S4 (109.1031E, 21.0700N)	MW489526	MW485227	MW485233
GCBG03	S4 (109.1031E, 21.0700N)	MW489527	MW485228	MW485234
GCBG04	S4 (109.1031E, 21.0700N)	MW489528	MW485229	MW485235
GCBG05	S7 (109.0844E, 21.0122N)	MW489529	MW485230	MW485236

Table S3. Primers and PCR product information.

rDNA Region	Primers	Primer Sequences	PCR Products	References
D1–D3 LSU	D1R	5′-ACCCGCTGAATTTAAGCATA-3′	1384-1395 bp	[1]
	28-1483R	5′-GCTACTACCACCACCAAGATCTGC-3′		
D8–D10 LSU	FD8	5′-GGATTGGCTCTGAGGGTTGGG-3′	889-949 bp	[2]
	RB	5′-GATAGGAAGAGCCGACATCGA-3′		
SSU	EukA	5′-AACCTGGTTGATCCTGCCAGT-3′	1617-1788bp	[3]
	EukB	5′- TGATCCTTCTGCAGGTTCACCTAC-3′		

References

1. Scholin, C.A.; Herzog, M.; Sogin, M.; Anderson, D.M. Identification of group and strain-specific genetic markers for globally distributed *Alexandrium* (Dinophyceae). II. Sequence analysis of a fragment of the LSU rRNA gene. *J. Phycol.* **1994,** *30*, 999–1011.
2. Chinain, M.; Faust, M.A.; Pauillac, S. Morphology and molecular analyses of three toxic species of *Gambierdiscus* (Dinophyceae): *G. pacificus*, sp. nov., *G. australes*, sp. nov., and *G. polynesiensis*, sp. nov. *J. Phycol.* **1999,** *35*, 1282–1296.
3. Medlin, L.; Elwood, H.J.; Stickel, S.; Sogin, M.L. The characterization of enzymatically amplified eukaryotic 16S-like rRNA-coding regions. *Gene* **1988,** *71*, 491–499.
